# Origin of the blood hyperserotonemia of autism

**DOI:** 10.1186/1742-4682-5-10

**Published:** 2008-05-22

**Authors:** Skirmantas Janušonis

**Affiliations:** 1 Department of Psychology, University of California, Santa Barbara, CA 93106-9660, USA

## Abstract

**Background:**

Research in the last fifty years has shown that many autistic individuals have elevated serotonin (5-hydroxytryptamine, 5-HT) levels in blood platelets. This phenomenon, known as the platelet hyperserotonemia of autism, is considered to be one of the most well-replicated findings in biological psychiatry. Its replicability suggests that many of the genes involved in autism affect a small number of biological networks. These networks may also play a role in the early development of the autistic brain.

**Results:**

We developed an equation that allows calculation of platelet 5-HT concentration as a function of measurable biological parameters. It also provides information about the sensitivity of platelet 5-HT levels to each of the parameters and their interactions.

**Conclusion:**

The model yields platelet 5-HT concentrations that are consistent with values reported in experimental studies. If the parameters are considered independent, the model predicts that platelet 5-HT levels should be sensitive to changes in the platelet 5-HT uptake rate constant, the proportion of free 5-HT cleared in the liver and lungs, the gut 5-HT production rate and its regulation, and the volume of the gut wall. Linear and non-linear interactions among these and other parameters are specified in the equation, which may facilitate the design and interpretation of experimental studies.

## Background

The blood hyperserotonemia of autism is an increase in the serotonin (5-hydroxytryptamine, 5-HT) levels in the blood platelets of a large subset of autistic individuals. It is usually reported as mean platelet 5-HT elevations of 25% to 50% in representative autistic groups [1] that almost invariably contain hyperserotonemic individuals. Since the first report in 1961 [2], this phenomenon has been described in autistic individuals of diverse ethnic backgrounds by many groups of researchers [3-9]. Despite the fact that the hyperserotonemia of autism is considered to be one of the most-well replicated findings in biological psychiatry [1], its biological causes remain poorly understood.

**Figure 1:**
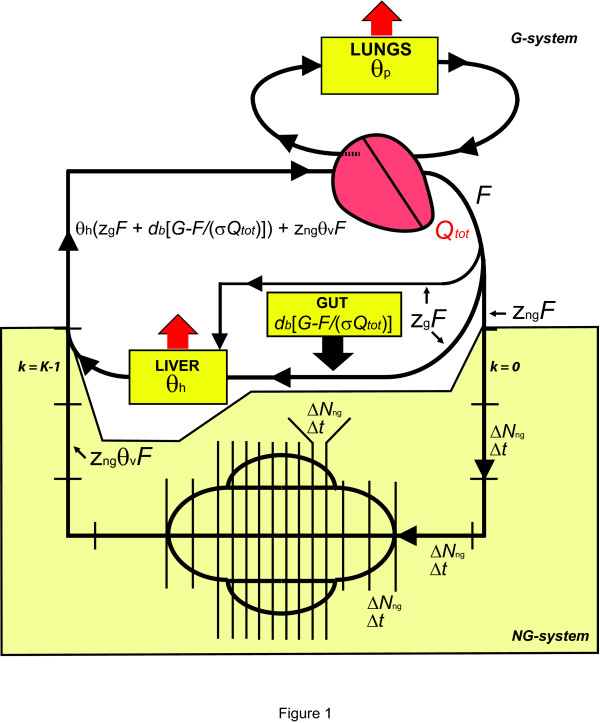
**The peripheral 5-HT circulation**. The thick black arrow represents the influx of 5-HT from the gut and the red arrows represent the clearance of 5-HT. For explanation of the variables, see the text, Table 1, and **Appendix 2**.

Blood platelets themselves do not synthesize 5-HT. During their life span of several days, they actively take up 5-HT from the blood plasma using a molecular pump, the 5-HT transporter (SERT). The plasma 5-HT originates in the gut, where most of it is synthesized by enterochromaffin cells (EC) of the gut mucosa [10]. Some of the gut 5-HT is used locally as a neurotransmitter of the enteric nervous system and it also can be taken up into gut cells that express SERT and low-affinity serotonin transporters [11,12]. Some of the gut 5-HT diffuses into the general blood circulation, where most of it is rapidly cleared by the liver and the lungs [13,14]. Free 5-HT in the blood plasma becomes available to platelets. The circulation of peripheral 5-HT is summarized in Figure 1.

The blood-brain barrier is virtually impermeable to 5-HT and, therefore, free 5-HT in the blood plasma is unlikely to reach cerebrospinal fluid or brain parenchyma. However, biological factors that cause the platelet hyperserotonemia may play a role in the early development of the autistic brain, since the brain and peripheral organs express many of the same neurotransmitter receptors and transporters. The consistency of the platelet hyperserotonemia suggests that many of the genes implicated in autism [15,16] may control a small number of functional networks. Since blood platelets are short-lived, the altered processes may remain active in the periphery years after the brain has formed. In contrast, most of the brain developmental processes are over by the time an individual is formally diagnosed with autism. SERT is expressed by brain neurons and blood platelets [17] and its altered function may both affect brain development and lead to abnormal 5-HT levels in platelets. To date, most experimental studies have focused on SERT polymorphisms as a likely cause of the platelet hyperserotonemia, but the results have been inconclusive. While SERT polymorphic variants may partially determine platelet 5-HT uptake rates [18] or even platelet 5-HT levels [19], these polymorphisms, alone, are unlikely to cause the platelet hyperserotonemia of autism [18,20]. Some evidence suggests that the platelet hyperserotonemia may be caused by altered 5-HT synthesis or release in the gut [21-23] or by interactions among several genes [24-26].

To date, most research into the causes of the platelet hyperserotonemia has focused on a specific part of the peripheral 5-HT system. However, this system is cyclic by nature and does not allow easy intuitive interpretation. It is not clear what parameters and their interactions platelet 5-HT levels are likely to be sensitive to, as well as what parameters should be controlled for when others are varied. For instance, an increase in SERT activity may increase platelet 5-HT uptake, but it may also increase 5-HT uptake in the gut and lungs and, consequently, may reduce the amount of free 5-HT in the blood plasma.

Here, we develop an equation that yields platelet 5-HT levels that are consistent with published experimental data. The equation also provides information about the sensitivity of platelet 5-HT levels to a set of biological parameters and their interactions.

## Results and Discussion

### Platelets take up 5-HT at low plasma 5-HT concentrations

Suppose blood platelets are produced at a constant rate, their half-life is *t*_1/2_, and we are interested in the steady state when the total number of platelets (*N*_*tot*_) remains constant. Then the number of the platelets whose age ranges from *x *≥ 0 to *x *+ *dx *is given by (**Appendix 1**)

(1) $$dN(x)=\frac{N_{tot}}{\tau }e^{-x/\tau }dx,$$

where *τ *= *t*_1/2_/ln 2 ≈ 1.44*t*_1/2_.

The 5-HT uptake rate of an "average" platelet at time *t *can be defined as follows:

(2) $$\bar{u}(t)\equiv \frac{1}{N_{tot}}\sum_{i=1}^{N_{tot}}u_{i}(t),$$

where *u*_*i*_(*t*) is the 5-HT uptake rate (mol/min) of platelet *i *at time *t*.

At any two times *t*_1 _and *t*_2 _(*t*_1 _≠ *t*_2_), at least some of the individual platelets in the circulation will be physically different, because platelets are constantly removed from the circulation and replaced by new platelets. Also, at least some individual platelets will be routed by the circulation to different blood vessels, which may have different concentrations of free 5-HT in the blood plasma. Since the platelet uptake rate depends on the 5-HT concentration in the surrounding plasma, generally, *u*_*i*_(*t*_1_) ≠ *u*_*i*_(*t*_2_). However, the 5-HT uptake and distribution of platelets appear to be little affected by their age or by how much 5-HT they have already accumulated [14,27]. Also, the numbers of platelets in blood vessels are very large and can be considered constant. Therefore, $\bar{u}(t)$ should be immune to these replacements and permutations, and the time-dependence of $\bar{u}(t)$ can be dropped:

(3) $$\bar{u}\equiv \bar{u}(t).$$

The total amount of 5-HT that has been taken up by the subpopulation of platelets whose age ranges from *x *to *x *+ *dx *is given by (**Appendix 1**)

(4) $$dU(x)=\frac{N_{tot}}{\tau }e^{-x/\tau }(\bar{u}x)dx.$$

If the total volume of the circulating blood is Ω_*b *_and the numerical concentration of platelets is *C*_*p *_= *N*_*tot*_/Ω_*b*_, the concentration of platelet 5-HT is

(5) $$C_{s}=\frac{1}{\Omega_{b}}\int_{0}^{\infty }dU(x)=\frac{1}{\Omega_{b}}\int_{0}^{\infty }\frac{N_{tot}}{\tau }e^{-x/\tau }(\bar{u}x)dx=\tau C_{p}\bar{u}.$$

It follows that

(6) $$\bar{u}=\frac{C_{s}}{\tau C_{p}}.$$

In normal humans, *C*_*s*_/*C*_*p *_has been experimentally estimated to be around 3.58 · 10^-18 ^mol/platelet [7]. The half-life of human platelets is approximately 5 days [28,29], so *τ *≈ 1.44*t*_1/2 _= 1.04 · 10^4 ^min. Plugging these values into Eq. (6) yields $\bar{u}$ = 3.44 · 10^-22 ^mol/min, or an "average" platelet takes up around 3.5 molecules of 5-HT every second.

What concentration of free 5-HT in the blood plasma corresponds to this uptake rate? Since platelet 5-HT uptake obeys Michaelis-Menten kinetics [14,18],

(7) $$u_{i}=\frac{V_{max}c_{i}}{K_{m}+c_{i}},$$

where *V*_*max *_is the maximal 5-HT uptake rate of one platelet, *K*_*m *_is the Michaelis-Menten constant, and *c*_*i *_is the local concentration of free 5-HT surrounding platelet *i*.

If the concentration of free 5-HT were the same in all blood vessels (*c*_*i *_≡ *C*_*f*_), we would obtain

(8) $$\bar{u}=\frac{V_{max}C_{f}}{K_{m}+C_{f}}$$

and

(9) $$C_{f}=\frac{\bar{u}K_{m}}{V_{max}-\bar{u}}.$$

However, in some blood vessels (such as the ones leaving the gut) the concentration of free 5-HT may be considerably higher than in others. We can define

(10) $$\bar{c}\equiv \frac{1}{N_{tot}}\sum_{i=1}^{N_{tot}}c_{i}$$

and rely on the evidence that *c*_*i *_≪ *K*_*m *_[14,18,30]. Then

(11) $$\bar{u}\approx \frac{V_{max}}{K_{m}}\bar{c}$$

and it follows that

(12) $$\bar{c}\approx \frac{K_{m}}{V_{max}}\bar{u}=\frac{K_{m}C_{s}}{\tau V_{max}C_{p}}.$$

In normal humans, *V*_*max *_≈ 1.26 · 10^-18 ^mol/(min · platelet) and *K*_*m *_≈ 0.60 · 10^-6 ^mol/L (these values were obtained by weighting the medians of each of the three groups of [18] by the number of subjects in the study). Plugging these values and the obtained $\bar{u}$ into Eq. (12) yields *C*_*f *_≈ $\bar{c}$ = 0.16 · 10^-9 ^mol/L = 0.16 nM.

Experimental measurement of free 5-HT in the blood plasma poses serious challenges. It is not uncommon to report concentration values of free 5-HT that are a few orders of magnitude higher than those obtained in carefully designed studies (for discussion, see [14,30,31]). The theoretically calculated value (0.16 nM) is on the same order as an accurate experimental estimate of free 5-HT in the distal venous plasma (0.77 nM) obtained by Beck et al. [30]. These authors note that new experimental methodologies may further reduce their estimate [30]. Taken together, these theoretical and experimental results suggest that virtually all platelets take up 5-HT at very low free 5-HT concentrations, after most of the 5-HT released by the gut has been cleared from the circulation by the liver and the lungs.

### Gut 5-HT release rate (*R*)

We denote the gut 5-HT release rate *R*, where *R *is expressed per unit volume of the gut wall and includes all 5-HT released by the gut. Specifically, *R *includes the 5-HT that (i) is taken back up into gut cells, (ii) remains in the extracellular space of the gut wall, and (iii) diffuses into the blood circulation. If the gut 5-HT release rate fluctuates but homeostatic mechanisms keep it near some constant value *R*_00 _> 0, then we can write

(13) $$\lambda \frac{dR}{dt}=R_{00}-R,$$

where *t *is time and *λ *> 0 is the time constant of the process (the larger is the *λ*, the slower is the return to *R*_00_). We next consider a more general scenario, where the gut 5-HT release rate is controlled by the actual state of the peripheral 5-HT system.

First, we consider a local mechanism that monitors the extracellular 5-HT concentration in the gut wall. The actual sensitivity of the gut 5-HT release rate to extracellular 5-HT levels is not well understood. In the brain raphe nuclei, 5-HT release does not appear to be controlled by 5-HT1A autoreceptors unless extracellular 5-HT levels become excessive [32]. The gut expresses 5-HT1A, 5-HT3, and 5-HT4 receptors [11], but these receptors may not be activated by the normal levels of endogenous extracellular 5-HT in the gut wall [33]. In SERT-deficient mice, 5-HT synthesis appears to be increased by around 50%, but the expression and activity of tryptophan hydroxylases 1 and 2 are not altered [34]. In SERT-deficient rats, the expression and activity of tryptophan hydroxylase 2 are also unaltered in the brain, even though the extracellular 5-HT levels in the hippocampus are elevated 9-fold [35]. From a systems-control perspective, the reported insensitivity of 5-HT synthesis to extracellular 5-HT levels may be due to the inherent ambiguity of the signal. In fact, high extracellular 5-HT levels may signal both overproduction of 5-HT by tryptophan hydroxylase and an excessive loss of presynaptic 5-HT due to its reduced uptake by SERT. If the former is the case, the activity of trypotophan hydroxylase should be decreased; if the latter is the case, it should be increased.

Alternatively, platelet 5-HT levels can be regulated by global peripheral mechanisms. Since platelets take up 5-HT over their life span, their 5-HT levels will change only if an alteration of the peripheral 5-HT system is sustained over a considerable period of time. Since platelets act as systemic integrators, we can assume that, *formally*, the gut 5-HT release rate is a function of the platelet 5-HT concentration. In essence, we simply assume that the gut 5-HT release is controlled by global, systemic changes in the peripheral serotonin system. In biological reality, this relationship would be mediated by latent variables, because platelet 5-HT is inaccessible to the gut.

If the gut release rate is controlled by any of the discussed mechanisms,

(14) $$\lambda \frac{dR}{dt}=R_{00}-R+f(G,P),$$

where *G *is the extracellular 5-HT concentration in the gut wall, *P *is the platelet 5-HT concentration (mol/platelet), and *f*(., .) is a differentiable function.

Linearization of *f*(*G, P*) in the neighborhood of "normal" values of *G *and *P *(denoted *G*_0 _and *P*_0_, respectively) yields

(15) *f*(*G*, *P*) = *f*(*G*_0_, *P*_0_) + *α*(*G*_0 _- *G*) + *β*(*P*_0 _- *P*),

where ${\alpha \equiv -\partial f/\partial G|}_{\left(G_{0},P_{0}\right)}\geq 0$ and ${\beta \equiv -\partial f/\partial P|}_{\left(G_{0},P_{0}\right)}\geq 0$.

By denoting *R*_0 _= *R*_00 _+ *f *(*G*_0_, *P*_0_) we obtain

(16) $$\lambda \frac{dR}{dt}=R_{0}-R+\alpha (G_{0}-G)+\beta (P_{0}-P).$$

Note that Eq. (13) is a special case of Eq. (16) when neither *G *nor *P *controls the gut 5-HT release rate (i.e., when *α *= *β *= 0).

### Concentration of extracellular 5-HT in the gut wall (*G*)

The concentration of extracellular 5-HT in the gut wall increases due to synthesis and release of 5-HT by EC cells and neurons of the gut. It decreases due to two processes: (i) local 5-HT uptake by SERT (and perhaps by other, low-affinity transporters [12,35]) and (ii) 5-HT diffusion into gut blood capillaries. Suppose that the blood that has exited the heart through the aorta at time *t *reaches the gut at time *t *+ *s *(*s *> 0). The decrease rate of extracellular 5-HT concentration in the gut wall due to the diffusion into blood capillaries is given, according to Fick's First Law, by

(17) $$\frac{DS}{w\Omega_{g}}\left[G(t+s)-\frac{z_{g}F(t)}{\sigma z_{g}Q_{tot}}\right]=d_{g}\left[G(t+s)-\frac{F(t)}{\sigma Q_{tot}}\right],$$

where *G*(*t *+ *s*) is the concentration of extracellular 5-HT in the gut wall at time *t *+ *s*, *D *is the 5-HT diffusion coefficient across the blood capillary wall, *S *is the total surface area of the gut blood capillaries, *w *is the thickness of the capillary wall, Ω_*g *_is the effective extracellular volume of the gut wall, *Q*_*tot *_is the total cardiac output, *z*_*g *_is the proportion of the total cardiac output routed to the gut and/or the liver, *F*(*t*) is the flow of free 5-HT in the aorta at time *t*, *σ *is the proportion of blood volume that is not occupied by cells (approximated well by 1 - *Ht*, where *Ht *is the hematocrit), and *d*_*g *_≡ *DS*/(*w*Ω_*g*_). Note that *z*_*g*_*F *(*t*)/(*σz*_*g*_*Q*_*tot*_) is the concentration of free 5-HT in the blood plasma that arrives in the gut at time *t *+ *s *(Fig. 1).

If all three discussed processes are taken into consideration,

(18) $$\frac{dG}{dt}=R(t)-k_{g}G(t)-d_{g}\left[G(t)-\frac{F(t-s)}{\sigma Q_{tot}}\right],$$

where *k*_*g *_is the 5-HT uptake rate constant in the gut wall. This constant is likely to be a function of SERT activity (*γ*), i.e., *k*_*g *_≡ *k*_*g*_(*γ*). Importantly, *k*_*g*_(0) is not necessarily zero, since 5-HT uptake in the gut may be mediated by low-affinity 5-HT transporters, at least in the absence of SERT [12,35].

### Flow of free 5-HT in the aorta (*F*)

We next consider the flow (mol/min) of free 5-HT in the blood circulation from the time blood exits the heart through the aorta (at time *t*) to the time it returns to the aorta after one circulation cycle (at time *t *+ *T*; Fig. 1). Since blood transit times from organ to organ are relatively short (seconds), we will ignore 5-HT diffusion parallel to the flow. After the blood leaves the heart, a proportion (*z*_*g*_) of the total cardiac output is routed to the gut and/or the liver. On arrival in the gut at time *t *+ *s *(0 <*s *<*T*), the blood is replenished with new 5-HT synthesized in the gut wall. According to Fick's First Law, this flow of 5-HT into the blood is

(19) $$\frac{DS}{w}\left[G(t+s)-\frac{z_{g}F(t)}{\sigma z_{g}Q_{tot}}\right]=d_{b}\left[G(t+s)-\frac{F(t)}{\sigma Q_{tot}}\right],$$

where all parameters and *G*(*t *+ *s*) are defined as in Eq. (17), *F*(*t*) is the flow of free 5-HT in the aorta, and *d*_*b *_≡ *DS*/*w *(note that *d*_*b*_/*d*_*g *_= Ω_*g*_).

After the 5-HT flow leaves the gut, it passes through the liver that removes a large proportion (1 - *θ*_*h*_) of free 5-HT [13,14]. After exiting the liver, the 5-HT flow is joined by the 5-HT flow that did not enter the gut and/or the liver and the merged flow passes through the lungs that remove another large proportion (1 - *θ*_*p*_) of free 5-HT [13,14]. Experimental results suggest that *θ*_*h *_≈ 0.25 and *θ*_*p *_≈ 0.08 [13]. Since the lungs express SERT [36], *θ*_*p *_may be considered to be a function of SERT activity, i.e., *θ*_*p *_≡ *θ*_*p*_(*γ*). It is likely that *θ*_*p*_(0)≠ 0, since no obvious toxic 5-HT effects are seen in mice that lack SERT [12].

Platelet 5-HT uptake is a slow process compared with the blood circulation through the gut, liver, and lungs. Therefore, in this circulation, platelet uptake should have a negligible effect on free 5-HT levels in the blood plasma [13,14]. However, platelets spend a considerable proportion of the circulation cycle in the vascular beds of other organs (the "non-gut" system of Fig. 1), where platelet 5-HT uptake may have an impact on the already low levels of free 5-HT.

Taking all these considerations together, the 5-HT flow that leaves the heart after one full circulation cycle is

(20) $$F(t+T)=\left[\left(z_{g}F(t)+d_{b}\left[G(t+s)-\frac{F(t)}{\sigma Q_{tot}}\right]\right)\theta_{h}+\theta_{v}z_{ng}F(t)\right]\theta_{p},$$

where 1 - *θ*_*v *_is the proportion of free 5-HT cleared by the platelets in the "non-gut" system (Fig. 1) and *z*_*ng *_= 1 - *z*_*g*_.

### Platelet 5-HT concentration at the steady state ($\overset{\wedge }{P}$)

Denote $\overset{\wedge }{F}$ the steady-state flow of free 5-HT in the aorta. The system is in its steady state if the following is true: *dR/dt *= 0, *dG/dt *= 0, *F*(*t*) = *F*(*t *- *T*) = $\overset{\wedge }{F}$, and if *F*(*t *- *s*) ≈ *F*(*t *- *s *- *x*) = $\overset{\wedge }{F}$ for all *x *> 0 for which *N*_*tot *_exp(-*x*/*τ*) ≫ 1, where 0 <*s *<*T *(for the last condition, see Eqs. (36) and (47) in **Appendix 2**).

At the steady state, the platelet 5-HT concentration is (**Appendix 2**)

(21) $$\overset{\wedge }{P}=\frac{\tau k_{p}\overset{\wedge }{F}}{\sigma Q_{tot}},$$

where *k*_*p *_≡ *k*_*p*_(*γ*) is the 5-HT uptake rate constant of one platelet. In mice lacking SERT, the amount of 5-HT stored in blood platelets in virtually zero [12], suggesting that *k*_*p*_(0) = 0.

Solving Eqs. (16), (18), (20), and (21) at the steady state yields

(22) $$\overset{\wedge }{P}=\frac{S_{1}+\beta P_{0}}{S_{2}+\beta },$$

where

(23) *S*_1 _= *R*_0 _+ *αG*_0_

and

(24) $$S_{2}=\frac{1}{\tau k_{p}}\left[\sigma Q_{tot}\Theta \left(\frac{K_{g}}{d_{b}}+\frac{1}{\Omega_{g}}\right)+K_{g}\right],$$

where for brevity we defined

(25) $$\Theta \equiv \frac{1-z_{g}\theta_{h}\theta_{p}-z_{ng}\theta_{v}\theta_{p}}{\theta_{h}\theta_{p}}$$

and

(26) *K*_*g *_≡ *k*_*g *_+ *α*.

In the derivation, we used the relationship *d*_*g *_= *d*_*b*_/Ω_*g*_.

**Figure 2:**
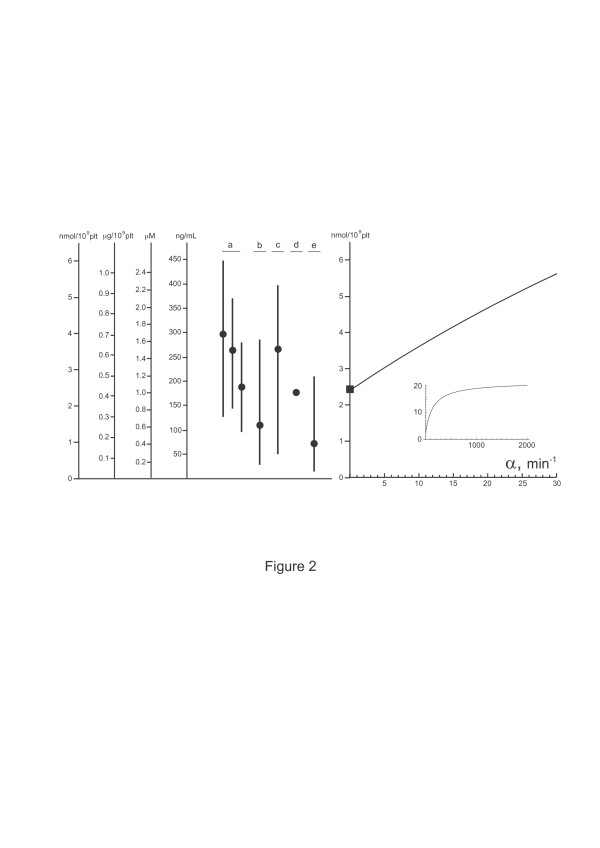
**Platelet 5-HT levels**. Normal platelet 5-HT concentrations reported in published reports (a [6], b [19], c [7], d [8], e [9]; the circles are the means and the bars indicate the range), compared with the theoretical values obtained with *α *= 0 (square) and *α *> 0. The values of the other parameters are given in Table 1 and *β *= 0. The theoretical platelet 5-HT concentrations reach a limit when *α *is large (inset).

The values of the parameters can be approximated based on published experimental results (Table 1). Since little is known about the regulation of 5-HT release from the gut, we can initially assume that *α *= *β *= 0 (in this case, platelet 5-HT concentration is independent of *G*_0 _and *P*_0_). Plugging the parameter values into Eq. (22) yields $\overset{\wedge }{P}$ = 2.40 · 10^-18 ^mol/platelet, or 4.23 · 10^-16^g/platelet. Since the platelet concentration in the blood has been estimated to be 4.28 · 10^8 ^platelets/mL [7], the obtained value is equivalent to the whole-blood 5-HT concentration of 1.02 *μ*M or 0.18 *μ*g/mL. These values are well within the range of normal 5-HT concentrations obtained in experimental studies (Fig. 2). Platelet 5-HT concentrations when *α *> 0 are plotted in Fig. 2.

**Table 1:** Parameter values

**Parameter**	**Value**	**Units**	**Source**	**Note**
		(plt = platelet)		
MW (5-HT)	176.22	g mol^-1^		1
*D*	6.00 · 10^-8^	m^2 ^min^-1^	[48]	2
*d*_*b*_	6.00	m^3 ^min^-1^	*d*_*b *_= *DS/w*	3
*d*_*g*_	5.82 · 10^3^	min^-1^	*d*_*g *_= *d*_*b*_/Ω_*g*_	4
*G*_0_	1.00 · 10^-6^	mol m^-3^	Table 1 of [32]	5
*k*_*g*_	4.00	min^-1^	Fig. 4A of [35]	6
*k*_*p*_	2.12 · 10^-15^	m^3 ^min^-1 ^plt^-1^	[18]	7
*R*_0_	1.65 · 10^-5^	mol m^-3 ^min^-1^	[14]	8
*P*_0_	3.58 · 10^-18^	mol plt^-1^	[7]	9
*S*	1.00 · 10^2^	m^2^	Table 8.3 of [48]	10
*Q*_*tot*_	5.60 · 10^-3^	m^3 ^min^-1^	[14]	11
*t*_1/2_	7.20 · 10^3^	min	[28, 29]	12
*w*	1.00 · 10^-6^	m	Table 8.2 of [48]	13
*z*_*g*_	0.27		Fig. 1 of [14]	14
*z*_*ng*_	0.73		*z*_*ng *_= 1 - *z*_*g*_	15
*α*	≥ 0	min^-1^	See note	16
*β*	≥ 0	plt m^-3 ^min^-1^	See note	17
*θ*_*h*_	0.25		[13]	18
*θ*_*p*_	0.08		[13]	19
*θ*_*v*_	0.50		[13]	20
*ρ*	9.70 · 10^4^	m^-1^	*ρ *= *S*/Ω_*g*_	21
*σ*	0.56		See note	22
*τ*	1.04 · 10^4^	min	*τ *= 1.44*t*_1/2_	23
Ω_*b*_	5.40 · 10^-3^	m^3^	Table 8.3 of [48]	24
Ω_*g*_	1.03 · 10^-3^	m^3^	[49]	25

1. The molecular weight of 5-HT (C_10_H_12_N_2_O).

2. The coefficient of 5-HT diffusion across the gut capillary wall. In liquids, the diffusion coefficient is on the order of 10^-5 ^cm^2^/s [48].

3. The rate constant of 5-HT influx into the blood due to 5-HT diffusion from the gut.

4. The rate constant of 5-HT loss in the gut due to 5-HT diffusion into the blood.

5. The homeostatic set point of the extracellular 5-HT concentration in the gut mucosa (irrelevant if *α *= 0). The concentration of extracellular 5-HT in the gut wall is unknown. We used an estimate based on extracellular 5-HT levels in the rat raphe nuclei [32]. Both the raphe nuclei and the gut mucosa synthesize 5-HT and express some of the same 5-HT receptors, such as the 5-HT1A receptor [32, 39].

6. The 5-HT uptake rate constant of the gut mucosa is unknown. We used an estimate based on measurements of 5-HT uptake in the normal (SERT +/+) rat brain [35]. The value of *V*_*max *_was assumed to be 4 pmol/min per milligram of protein [35], the protein content in the brain was assumed to be 10% (w/w) [50], and the specific weight of fresh brain tissue was 1 g/mL [51]. This yielded *V*_*max *_= 4 · 10^-4 ^mol/min per cubic meter of fresh tissue. The value of *K*_*m *_was assumed to be 100 nmol/L [35]. Since *K*_*m *_is much larger than the extracellular 5-HT concentration [32], *k*_*g *_was calculated as *V*_*max*_/*K*_*m*_. (As this article was being prepared for publication, Gill et al. [52] published a detailed report on the expression and kinetics of the human gut SERT.)

7. The 5-HT uptake rate constant of one platelet. The *V*_*max *_and and *K*_*m *_values were obtained by weighting the medians of each of the three groups of [18] by the number of subjects in the study. Since *K*_*m *_is much larger than the concentration of free 5-HT in the blood plasma [14], *k_p_* was calculated as *V*_*max*_/*K*_*m*_.

8. The gut 5-HT release rate that is independent of both the extracellular 5-HT concentration in the gut wall and the platelet 5-HT concentration. The gut 5-HT production estimate of 3000 ng/min was used [14]. In order to obtain the 5-HT release rate per unit volume of the gut wall (*R*_0_), this estimate was divided by Ω_*g *_and further assumed to be independent of Ω_*g*_.

9. The homeostatic set point for the platelet 5-HT concentration (irrelevant if *β *= 0).

10. The total surface area of blood capillaries in the gut was assumed to be on the order of 10^8 ^mm^2^, since the total surface of the body capillaries has been estimated to be 2.98 · 10^8 ^mm^2 ^[48].

11. The total cardiac output.

12. The half-life of blood platelets.

13. The wall thickness of blood capillaries in the gut.

14. The proportion of the total cardiac output routed to the gut and/or the liver (Fig. 1).

15. The proportion of the total cardiac output not routed to the gut and/or the liver (Fig. 1).

16. The gain of the regulation of the gut 5-HT release rate that is controlled by extracellular 5-HT concentration in the gut wall.

17. The gain of the regulation of the gut 5-HT release rate that is controlled by platelet 5-HT concentration.

18. One minus the proportion of free 5-HT in the blood plasma that is removed by the liver in one cycle of blood circulation. Based on an estimate obtained in the dog [13].

19. One minus the proportion of free 5-HT in the blood plasma that is removed by the lungs in one cycle of blood circulation. Based on an estimate obtained in the dog [13].

20. One minus the proportion of free 5-HT in the blood plasma that is removed in the "non-gut" (NG) system (Fig. 1) in one cycle of blood circulation. Based on estimates obtained in the dog [13].

21. The surface area of blood capillaries per unit volume of the gut mucosa.

22. The proportion of blood volume not occupied by cells. It is approximated well by 1 - *Ht*, where *Ht *= 0.44 is the hematocrit.

23. The time constant of platelet removal from the blood circulation.

24. The total volume of the circulating blood.

25. The total volume of the gut wall. Since EC cells are distributed from the stomach through the colon [10], the gut was assumed to be a cylinder with a length of 8 m and a diameter of 4 cm. The gut mucosa contains both the main source of peripheral 5-HT (the EC cells) and a dense meshwork of blood capillaries [53]. Therefore, the effective width of the gut wall was considered to be equal to the average length of the villi of the mucosa, or around 1 mm [49].

### Sensitivity of platelet 5-HT to parameters

Equation (22) represents the minimal set of relationships that have to be taken into account in experimental studies. It provides information about the sensitivity of platelet 5-HT levels to biological parameters and their interactions, some of which have not been considered or controlled for in experimental approaches. Here, we limit sensitivity analysis to the simplest case when parameters in Eq. (22) can be considered independent.

First, we calculate the local rate of change in $\overset{\wedge }{P}$ with respect to each of the parameters, i.e., we evaluate the partial derivatives of $\overset{\wedge }{P}$ with respect to each of the parameters at the parameter values given in Table 1 (see **Appendix 3 **for details). We express this rate of change as the percentage-wise change in $\overset{\wedge }{P}$ if a parameter increases by 10% with respect to its normal value (assuming the relationship can be approximated as linear). The results of these calculations are given in Table 2.

**Table 2:** Sensitivity of platelet 5-HT concentration to changes in parameters

**Parameter**, Δ = +10%	**Platelet 5-HT**, Δ%	**Platelet 5-HT**, Δ%
	*α *= 0 min^-1^	*α *= 20 min^-1^
	*β *= 0	*β *= 0
*d*_*b*_	6.7 · 10^-3^	3.5 · 10^-2^
*G*_0_	0	5.5
*k*_*g*_	-0.27	-0.24
*k*_*p*_	10.0	10.0
*R*_0_	10.0	4.5
*P*_0_	0	0
*Q*_*tot*_	-9.7	-8.6
*α*	0	4.3
*θ*_*h*_	9.8	8.6
*θ*_*v*_	0.29	0.26
*θ*_*p*_	10.1	8.9
*σ*	-9.7	-8.6
*τ*	10.0	10.0
Ω_*g *_(*S *constant)	9.7	8.6
Ω_*g *_(*ρ *constant)	9.7	8.6

The change in the normal platelet 5-HT concentration (%) if a parameter is increased by 10% with respect to its normal value given in Table 1. The relationship between the parameter and the platelet concentration is assumed to be linear for this small change (see **Appendix 3 **for details). All the other parameters are held constant at the values given in Table 1.

Second, we inverse the problem and calculate the percentage-wise change in each of the parameters needed to reach a 25% or 50% increase in platelet 5-HT concentration. These increases represent the typical range of elevation in platelet 5-HT levels in autism [1]. The required changes of the parameters are calculated using Eq. (22) without linearization. The results of these calculations are given in Table 3.

**Table 3:** Parameter changes causing 25% and 50% increases in platelet 5-HT concentration

	$\overset{\wedge }{P}$ + 25%	$\overset{\wedge }{P}$ + 25%	$\overset{\wedge }{P}$ + 50%	$\overset{\wedge }{P}$ + 50%
**Parameter**	**Value**	Δ, %	**Value**	Δ, %

*d*_*b*_	DNE	DNE	DNE	DNE
*G*_0_	DNE	DNE	DNE	DNE
*k*_*g*_	DNE	DNE	DNE	DNE
*k*_*p*_	2.65 · 10^-15^	25	3.18 · 10^-15^	50
*R*_0_	2.06 · 10^-5^	25	2.48 · 10^-5^	50
*P*_0_	DNE	DNE	DNE	DNE
*Q*_*tot*_	4.45 · 10^-3^	-21	3.68 · 10^-3^	-34
*α*	4.80	-	9.92	-
*θ*_*h*_	0.31	26	0.38	52
*θ*_*v*_	DNE	DNE	DNE	DNE
*θ*_*p*_	0.10	25	0.12	49
*σ*	0.44	-21	0.37	-34
*τ*	1.30 · 10^4^	25	1.56 · 10^4^	50
Ω_*g *_(*S *constant)	1.30 · 10^-3^	26	1.57 · 10^-3^	52
Ω_*g *_(*ρ *constant)	1.30 · 10^-3^	26	1.57 · 10^-3^	52

All units are the same as in Table 1. Unless *α *is varied, *α *= *β *= 0. When *α *is varied, its initial value is zero and *β *= 0. For each parameter, the other parameters are held constant at the values given Table 1. DNE = the required value does not exist.

Tables 2, 3 indicate that platelet 5-HT concentration is highly sensitive to the platelet 5-HT uptake rate constant (*k*_*p*_), the baseline gut 5-HT release rate (*R*_0_), the proportion of 5-HT cleared in the liver and lungs (*θ*_*h*_, *θ*_*p*_), and the volume of the gut wall (Ω_*g*_). Some experimental evidence suggests that *k*_*p *_is altered in autistic individuals [37,38]. The analysis also suggests that the hyperserotonemia of autism may be caused by altered extracellular 5-HT-dependent regulation of the gut release rate (*α*). We have recently shown that mice lacking the 5-HT1A receptor, expressed in the gut [39], develop an autistic-like blood hyperserotonemia [23], which may be caused by altered regulation of the gut 5-HT release rate. Another potentially important 5-HT receptor is the 5-HT4 receptor that is expressed throughout the gastrointestinal tract in humans [40]. The analysis also shows that the 5-HT uptake rate constant in the gut wall (*k*_*g*_) and the rate constant of 5-HT diffusion into the blood (*d*_*b*_) should have little effect on platelet 5-HT levels. A recent study has found no link between platelet hyperserotonemia and increased intestinal permeability in children with pervasive developmental disorders [41].

In the analysis we assumed that each parameter can be manipulated independently of the other parameters. In particular, this assumes that Ω_*g *_can be changed independently of *d*_*b*_, which is a function of *S*, the capillary surface area of the gut. However, increasing Ω_*g *_is likely to increase *S*. To make Ω_*g *_and *d*_*b *_truly independent, it is sufficient to make an assumption that a unit volume of the gut wall contains a constant surface area of blood capillaries, i.e., *S*/Ω_*g *_≡ *ρ *= const. Since *d*_*b *_= *DS/w*, this yields

(27) $$S_{2}=\frac{1}{\tau k_{p}}\left[\frac{\sigma Q_{tot}}{\Omega_{g}}\Theta \left(\frac{wK_{g}}{D_{\rho }}+1\right)+K_{g}\right].$$

After this correction, the sensitivity of platelet 5-HT concentration to the gut volume remains virtually unchanged (Tables 2, 3).

Care should be exercised in manipulating the parameters *k*_*p*_, *k*_*g*_, *θ*_*p*_, and *θ*_*v*_, which may not be independent. All of them may be determined, at least in part, by SERT activity (*γ*). Given the lack of experimental data regarding their actual relationships, two extreme scenarios can be considered. As assumed in the sensitivity analysis, these parameters can be considered to be virtually independent, since each of them is likely to be determined (in addition to SERT) by other factors in the platelet, gut, and lungs. Alternatively, all four parameters may be functions of only one variable, γ. In this case, platelet 5-HT levels may increase or decrease with different γ values, even if each of the functions were linear. This behavior of $\overset{\wedge }{P}$ as a function of *γ *is important to consider in SERT polymorphism studies. The ambiguity could be resolved if an experimentally-obtained covariance matrix for *k*_*p*_, *k*_*g*_, *θ*_*p*_, and *θ*_*v *_were available. Equation (22) also suggests that platelet 5-HT levels may be highly sensitive to interactions among the platelet uptake rate, the proportion of 5-HT cleared in the liver and lungs, the gut 5-HT release rate, and the volume of the gut wall. The length of the human gut is known to be remarkably variable [42], which may underlie some variability in platelet 5-HT levels. This possibility has not been investigated experimentally or theoretically. It is worth noting that 5-HT itself plays important roles in gastrulation [43] and morphogenesis [44], and that changes in gut length may have had a major impact on the evolution of the human brain [45].

It should be noted that Eq. (22) remains valid if some or all of the parameters are expressed as functions of new, independent parameters. In this case, the original parameters may no longer be independent and changing one of the new parameters may alter more than one of the original parameters. For instance, serotonin uptake in blood platelets has been recently shown to be dependent on interaction between SERT and integrin *α*IIb*β*3 [46]. Denoting the activity of the integrin *y*, we can write *k*_*p *_= *k*_*p*_(*γ*, *y*). It is possible that some other parameters in Eq. (22) can also be expressed as functions of integrin *α*IIb*β*3 activity. All of these functions can be plugged into Eq. (22), which remains to be correct and now allows calculation of platelet concentration as a function of integrin *α*IIb*β*3 activity, i.e., $\overset{\wedge }{P}$ = $\overset{\wedge }{P}$(*y*). Generally, further theoretical progress will largely depend on understanding the relationships among the current set parameters. Whether they can be expressed as functions of a smaller set of parameters is not known.

### Assumptions and caveats

Many of the assumptions in the model are "natural" in the sense that they are commonly used to explain experimental results (even though they may not be explicitly stated). In essence, the model simply formalizes the idea that peripheral 5-HT is produced in the gut, from which it can diffuse into the systemic blood circulation, where it can be transported into blood platelets. The strength of the model is in its "bird's-eye" view of the entire system. In particular, the model does not allow focusing on one parameter without explicitly stating what assumptions are made regarding the other parameters (some of which may be equally important in determining platelet 5-HT levels). For example, studies on SERT polymorphisms often focus on 5-HT uptake in platelets but do not explain how the same polymorphisms may affect 5-HT release from the gut (which also expresses SERT). The model also indicates which parameters and their interactions platelet 5-HT concentration is likely to be sensitive to, thus limiting one's freedom in choosing which factors can fall "outside the scope" of a study. By its very nature, the platelet hyperserotonemia of autism is a systems problem.

Some of the model assumptions are not critical, such as the assumption that the gut 5-HT release rate can be controlled by extracellular 5-HT in the gut wall or by platelet 5-HT levels. In the model, the absence of control is simply a special case of this more general scenario, since we can always set *α *= *β *= 0. If control is present, the assumption of its linearity (Eq. (16)) is necessary to obtain Eq. (22). While the Taylor series, used in Eq. (15), guarantees near-linear behavior of the control mechanisms in the neighborhood of *G*_0 _and *P*_0_, nothing is said about how far one can move away from *G*_0 _and *P*_0 _before non-linearities can no longer be ignored.

The assumption of the independence of the parameters in Eq. (22) is not necessary and is used here only to simplify the numerical sensitivity analysis. Some or all of the parameters may be tightly linked, which does not change Eq. (22) (but it may change the results obtained in the sensitivity analysis). Interdependent parameters can be expressed as functions of other, independent parameters (or "parameterized" in the mathematical sense), and these functions can be substituted for the parameters in Eq. (22). In this case, $\overset{\wedge }{P}$ becomes a function of the new parameters, as already discussed with regard to integrin *α*IIb*β*3.

The model assumes that the gut 5-HT release rate is constant at the steady-state. Strictly speaking, this assumption is incorrect, since gut activity exhibits circadian and other rhythmic behavior. Likewise, platelet counts exhibit normal fluctuations due to a number of factors, such as exercise, digestion, exposure to ultraviolet light, and others [47]. However, platelets accumulate 5-HT over days; therefore, *R *and *N*_*tot *_can be thought of as "baseline" values.

A potentially important assumption is made regarding the nature of the 5-HT diffusion from the gut into the blood circulation. Passive diffusion is assumed, and the value of the diffusion coefficient (*D*) is considered to be comparable to typical values observed in liquids. Virtually no experimental data are available on the exact nature of the 5-HT diffusion (which may be facilitated), and its *D *value remains to be determined.

A set of critical assumptions limits relationships between the parameters (given in Table 1), which are assumed to be constant in an individual, and the four dynamic variables (*R*, *G*, *F*, and *P*), which can evolve in time. While any parameter can be a function of any other parameters, original or new, none of the parameters (original or new) can be a function of any of the dynamic variables. If this condition is not met, the steady-state platelet 5-HT concentration will have a form different from Eq. (22). Suppose extracellular 5-HT in the gut wall controls SERT expression, or free 5-HT in the blood plasma controls the proportion of internalized SERT in blood platelets [24]. In these cases, the model may fail because the uptake rate constants will depend on the dynamic variables, i.e., *k*_*g *_= *k*_*g*_(*G*(*t*)) and *k*_*p *_= *k*_*p*_(*F *(*t*)). Likewise, Eqs. (16), (18), (20), and (21) are assumed to exhaust all relationships between the four dynamic variables. If, for instance, Eq. (16) were changed to

(28) $$\lambda \frac{dR}{dt}=R_{0}-R+\alpha (G_{0}-G)+\beta (P_{0}-P)+\beta^{\prime }(F_{0}-F),$$

where *F*_0 _and *β*' are constants and *β*' ≠ 0, the solution in Eq. (22) would no longer be correct.

These critical assumptions define the limits within which the model should perform reasonably well. New experimental data will be needed to further refine it.

## Conclusion

We developed an equation that allows calculation of platelet 5-HT levels as a function of biological parameters. While the main goal is to understand the origin of the hyperserotonemia of autism, the equation can also be used to calculate platelet 5-HT levels in normal individuals and in individuals whose peripheral 5-HT system may be altered due to conditions unrelated to autism. In the simplest case when each parameter is manipulated independently, theoretical analysis predicts that platelet 5-HT concentration should be sensitive to changes in the platelet 5-HT uptake rate constant, the proportion of free 5-HT cleared in the liver and lungs, the gut 5-HT production rate and its regulation, and the volume of the gut wall. The equation also specifies linear and non-linear interactions among these and other parameters, some of which may also play a role in the developing autistic brain.

## Methods

All symbolic and numerical calculations were done in Mathematica 6.0.1 (Wolfram Research, Inc.). For convenience, symbols used in the text are listed in Table 4.

**Table 4:** Symbols used in the text

**Symbol**	**Definition**
*C*_*p*_	Numerical concentration of platelets in the blood
*C*_*s*_	Amount of 5-HT per platelet (platelet 5-HT concentration)
*D*	Diffusion coefficient of 5-HT diffusion from the gut wall into gut blood capillaries
*d*_*b*_	Rate constant of 5-HT influx into the blood due to 5-HT diffusion from the gut
*d*_*g*_	Rate constant of 5-HT loss in the gut due to 5-HT diffusion into the blood
*F *= *F*(*t*)	Flow of free 5-HT in the aorta as a function of time
$\overset{\wedge }{F}$	Steady-state flow of free 5-HT in the aorta
*G*_0_	Theoretical concentration of extracellular 5-HT in the gut wall around which the control of gut 5-HT release is near-linear
*G *= *G*(*t*)	Extracellular 5-HT concentration in the gut wall as a function of time
$\overset{\wedge }{G}$	Steady-state extracellular 5-HT concentration in the gut wall
*k*_*g*_	5-HT uptake rate constant in the gut wall
*k*_*p*_	5-HT uptake rate constant in blood platelets
*N*_*tot*_	Total number of platelets
*R*_0_	Theoretical, steady-state gut 5-HT release rate achieved when *α *= *β *= 0
*R *= *R*(*t*)	Gut 5-HT release rate as a function of time
$\overset{\wedge }{R}$	Steady-state gut 5-HT release rate
*P*_0_	Theoretical 5-HT concentration in blood platelets around which the control of gut 5-HT release is near-linear
$\overset{\wedge }{P}$	Steady-state platelet 5-HT concentration
*S*	Total surface of the blood capillaries in the gut wall
*Q*_*tot*_	Total cardiac output
*t*	Time
*t*_1/2_	Half-life of blood platelets
$\bar{u}$	5-HT uptake rate of an "average" blood platelet
*T*	Period of blood circulation
*w*	Wall thickness of gut capillaries
*z*_*g*_	Proportion of cardiac output routed to the gut and/or liver
*z*_*ng*_	Proportion of cardiac output not routed to the gut and/or liver
*α*	Gain of the gut 5-HT release control that monitors the extracellular 5-HT concentration in the gut wall
*β*	Gain of the gut 5-HT release control that monitors the 5-HT concentration in platelets
*γ*	SERT activity
*θ*_*h*_	1- proportion of free 5-HT cleared by the liver in one blood circulation cycle (Fig. 1)
*θ*_*p*_	1- proportion of free 5-HT cleared by the lungs in one blood circulation cycle (Fig. 1)
*θ*_*v*_	1- proportion of free 5-HT cleared by the vascular beds of the "non-gut" system (Fig. 1)
*ρ*	Surface area of blood capillaries per unit volume of the gut wall
*σ*	Proportion of blood volume not occupied by cells
*τ*	Time constant of the decay of platelet numbers due to their aging
Ω_*b*_	Total volume of circulating blood
Ω_*g*_	Total volume of the gut wall

Only symbols used throughout the text are listed. Their precise definitions are given in the text.

## Authors' contributions

SJ developed the model and wrote the manuscript.

## Appendix

### 1. Distribution of blood platelets by age

To derive Eqs. (1) and (4), consider the platelets whose age is between *x *= *k*Δ*x *and *x *+ Δ*x*, where Δ*x *> 0 is small and *k *= 0, 1, 2... If the platelet production rate is denoted *r*, the total number of platelets produced in the interval *x *is *r*Δ*x*. With each time step Δ*x*, this number decreases by a factor of *q*, where *q *= *e*^-Δ*x*/*τ *^(this follows directly from the fact that the decay of platelet numbers can be described by a constant half-life). The number of the remaining platelets after *k *time steps is given by

(29) Δ*N*(*k*) = (*r*Δ*x*)*q*^*k*^.

The total number of platelets currently circulating in the blood then is

(30) $$N_{tot}=\sum_{k=0}^{\infty }\Delta N(k)=r\Delta x\sum_{k=0}^{\infty }q^{k}=\frac{r\Delta x}{1-q}.$$

It follows from Eqs. (29) and (30) that

(31) Δ*N*(*k*) = *N*_*tot*_(1 - *q*)*q*^*k *^= *N*_*tot*_(1 - *e *^-Δ*x*/*τ*^)*e*^-*k*Δ*x*/*τ*^.

Since Δ*x *≪ *τ *and

(32) $$1-e^{-\Delta x/\tau }=\frac{\Delta x}{\tau }-\frac{{\left(\Delta x\right)}^{2}}{2\tau^{2}}+\frac{{\left(\Delta x\right)}^{3}}{6\tau^{3}}-\ldots ,$$

we obtain

(33) $$\Delta N(k)\approx \frac{N_{tot}\Delta x}{\tau }e^{-k\Delta x/\tau }.$$

Then the platelets whose age is between *x *and *x *+ Δ*x *have taken up the following amount of 5-HT:

(34) $$\Delta U(k)=\Delta N(k)(\bar{u}k\Delta x)\approx \frac{N_{tot}\Delta x}{\tau }e^{-k\Delta x/\tau }(\bar{u}k\Delta x),$$

where $\bar{u}$ is the 5-HT uptake rate of an "average" platelet, defined in Eqs. (2) and (3). If Δ*x *is allowed to tend to zero, Eqs. (33) and (34) become Eqs. (1) and (4).

### 2. Platelet 5-HT concentration

Consider the circulation of peripheral 5-HT (Fig. 1). We start by dividing the peripheral 5-HT system into the "gut" system (G-system) and the "non-gut" system (NG-system). In the G-system, arterial blood exits the heart through the aorta, perfuses the gut and/or the liver, joins the venous blood flow to the heart, passes through the lungs, and returns to the heart with the oxygenated blood. In the NG-system, arterial blood exits the heart through the aorta, perfuses various peripheral organs, and joins the venous blood flow. In further considerations, the blood flow rate (measured in m^3^/min) is clearly distinguished from the 5-HT flow rate (measured in mol/min). In fact, if a blood vessel carrying 5-HT-enriched blood is joined by another blood vessel with virtually no 5-HT in its blood, the blood flow rate of the merged vessel increases, but its 5-HT flow rate remains the same. We intentionally avoid the term "flux", which often denotes flow rate per unit area.

Denote *Q*_*tot *_the total cardiac output, *z*_*ng *_the proportion of the cardiac output that does not pass through the gut and/or the liver, and *N*_*tot *_the total number of blood platelets in the circulation. Then the blood flow rate of the NG-system is *Q*_*ng *_= *z*_*ng*_*Q*_*tot *_and at any time the NG-system contains *N*_*ng *_= *ηN*_*tot *_uniformly distributed platelets (0 ≤ *z*_*ng*_, *η *≤ 1). If every platelet passing through the NG-system travels an approximate linear distance *L*, we can subdivide *L *into *K *(not necessarily equal) segments, each of which contains the same number of platelets Δ*N*_*ng *_= *ηN*_*tot*_/*K *(Fig. 1). Assuming these groups of platelets advance in discrete time steps, each of them will spend the same constant time, Δ*t*, in each of the linear segments:

(35) $$\Delta t=\frac{\Delta N_{ng}}{Q_{ng}C_{p}},$$

where *C*_*p *_is the concentration of platelets in the blood (the number of platelets per unit volume of blood). Denote *F*(*t*) the flow of free 5-HT that exits the heart through the aorta at time *t*. Next, consider Δ*N*_*ng *_platelets that exit the heart through the aorta at time *t *and enter the NG-system at time *t *+ *s *(*s *> 0). The flow of free 5-HT that enters the NG-system with these platelets at time *t *+ *s *is *z*_*ng*_*F *(*t*). The concentration of free 5-HT around these platelets at time *t *+ *s *then is

(36) $$c_{ng}(t+s)=\frac{z_{ng}F(t)}{\sigma Q_{ng}}=\frac{F(t)}{\sigma Q_{tot}},$$

where *σ *is the proportion of blood volume not occupied by cells.

If the 5-HT uptake rate constant of one platelet is *k*_*p*_, the total 5-HT amount taken up by the NG-system platelets from time *t*_1 _to *t*_1 _+ Δ*t *is

(37) $$U_{ng}(t_{1},t_{1}+\Delta t)=\sum_{k=0}^{K-1}\frac{\eta N_{tot}}{K}c_{ng}(t_{1}-k\Delta t)k_{p}\Delta t.$$

Next, consider a time period from *t*_1 _to *t*_2 _= *t*_1 _+ *M*Δ*t *(*M *= 2, 3...). During this time, the total amount of 5-HT taken up by the NG-system platelets is

(38) $$U_{ng}(t_{1},t_{2})=\sum_{m=0}^{M-1}\sum_{k=0}^{K-1}\frac{\eta N_{tot}}{K}c_{ng}(t_{1}+(m-k)\Delta t)k_{p}\Delta t.$$

If *M *≥ *K*,

(39) $$U_{ng}(t_{1},t_{2})=\sum_{m=0}^{M-1}K\frac{\eta N_{tot}}{K}c_{ng}(t_{1}+m\Delta t)k_{p}\Delta t+(\delta_{1}-\delta_{2}),$$

where

(40) $$\delta_{1}=\sum_{k=1}^{K-1}k\frac{\eta N_{tot}}{K}c_{ng}(t_{1}+(k-K)\Delta t)k_{p}\Delta t$$

and

(41) $$\delta_{2}=\sum_{k=M-K+1}^{M-1}(k-M+K)\frac{\eta N_{tot}}{K}c_{ng}(t_{1}+k\Delta t)k_{p}\Delta t.$$

Since blood platelets accumulate 5-HT over a period of time that is a few orders of magnitude longer than one blood circulation cycle, we are interested in the situation when *M *≫ *K*. Then, if *F*(*t*) satisfies mild constraints (e.g., does not fluctuate rapidly), *δ*_1 _and *δ*_2 _can be dropped and Eq. (39) becomes

(42) $$U_{ng}(t_{1},t_{2})\approx \eta k_{p}N_{tot}\sum_{m=0}^{M-1}c_{ng}(t_{1}+m\Delta t)\Delta t.$$

If we allow Δ*t *to tend to zero,

(43) $$U_{ng}(t_{1},t_{2})\approx \eta k_{p}N_{tot}\int_{t_{1}}^{t_{2}}c_{ng}(x)dx.$$

Thus far, we have ignored the fact that blood platelets are destroyed and replaced by new platelets. However, the half-life of platelets is only approximately 5 days [28,29]. Consider a past time *t*_0 _(*t*_0 _<*t*, where *t *is present time). Among the presently circulating platelets, the proportion of the platelets that are older than *t *- *t*_0_, according to Eq. (1), is

(44) $$\frac{1}{N_{tot}}\int_{t-t_{0}}^{\infty }\frac{N_{tot}}{\tau }e^{-x/\tau }dx=e^{-(t-t_{0})/\tau }.$$

Only these platelets were taking up 5-HT when the concentration of free 5-HT in the blood entering the NG-system at time *t*_0 _was *c*_*ng*_(*t*_0_). Therefore, the total 5-HT amount accumulated by the NG-system platelets at time *t *can be found by weighting the past concentrations of free 5-HT by the proportion of the presently circulating platelets that were taking up 5-HT at these past times:

(45) $$U_{ng}(t)\approx \eta k_{p}N_{tot}\int_{-\infty }^{t}c_{ng}(x)e^{-(t-x)/\tau }dx,$$

where *U*_*ng*_(*t*) ≡ *U*_*ng*_(-∞, *t*).

After changing the dummy variable under the integral sign, we obtain

(46) $$U_{ng}(t)\approx \eta k_{p}N_{tot}\int_{0}^{\infty }c_{ng}(t-x)e^{-x/\tau }dx.$$

At a steady state,

(47) $$c_{ng}(t-x)\approx \bar{c_{ng}}$$

for all *x *for which *N*_*tot *_exp(-*x*/*τ*) ≫ 1 (i.e., more than one currently circulating platelet was produced before time *t -x*). Then the steady-state amount of 5-HT accumulated by the platelets of the NG-system is

(48) $$\bar{U_{ng}}\approx \eta k_{p}N_{tot}\tau \bar{c_{ng}}.$$

By analogy, the 5-HT accumulated by the platelets of the G-system at the steady state is expected to be

(49) $$\bar{U_{g}}\approx \sum_{i}\mu_{i}(1-\eta )k_{p}N_{tot}\tau \bar{c_{g,i}},$$

where $\bar{c_{g,i}}$ is the steady-state concentration of free plasma 5-HT in the *i*th compartment of the G-system, *μ*_*i*_> 0, and Σ_*i*_*μ*_*i *_= 1. Then

(50) $$\bar{U_{g}}\approx (1-\eta )k_{p}N_{tot}\tau \bar{c_{g}},$$

where $\bar{c_{g}}$ is the mean of the steady-state concentrations of free 5-HT in the compartments of the G-system:

(51) $$\bar{c_{g}}\equiv \sum_{i}\mu_{i}\bar{c_{g,i}}.$$

We have already shown that virtually all platelets take up 5-HT at very low free 5-HT concentrations. This is not surprising, since the blood that has left the gut reaches the liver within seconds [13,14], and the liver removes more than 70% of free 5-HT [13]. Assuming $\bar{c_{g}}\approx \bar{c_{ng}}$, we obtain the total amount of 5-HT accumulated by all blood platelets of the peripheral 5-HT system at the steady state:

(52) $$\overset{\wedge }{U}=\bar{U_{ng}}+\bar{U_{g}}\approx k_{p}N_{tot}\tau \bar{c_{ng}}.$$

The concentration of platelet 5-HT at the steady state then is

(53) $$\overset{\wedge }{P}=\frac{\overset{\wedge }{U}}{N_{tot}}\approx k_{p}\tau \bar{c_{ng}}.$$

Since, according to Eq. (36),

(54) $$\bar{c_{ng}}=\frac{\overset{\wedge }{F}}{\sigma Q_{tot}},$$

where $\overset{\wedge }{F}$ is the steady-state flow of free 5-HT in the aorta, we obtain

(55) $$\overset{\wedge }{P}\approx \frac{\tau k_{p}\hat{F}}{\sigma Q_{tot}}.$$

For the convenience of notation, we will further consider Eq. (55) to be exact.

### 3. Sensitivity of platelet 5-HT levels to changes in parameters

We investigate the sensitivity of $\overset{\wedge }{P}$ to changes in the parameters, which for the purpose of this analysis are considered to be independent. For the convenience of notation, we denote the set of parameters in Eq. (22) **X **= (*X*_1_, *X*_2_, *X*_3_, *X*_4_, ...) ≡ (*α*, *β*, *k*_*g*_, *k*_*p*_, ...), $\overset{\wedge }{P}$ (**X**) ≡ $\overset{\wedge }{P}$. Two approaches are used.

In the first approach, for each parameter *X*_*i *_we calculate the normalized differential

(56) $$\frac{d\overset{\wedge }{P}(X^{*},\Delta X_{i})}{\overset{\wedge }{P}(X^{*})}\times 100\%=\frac{\Delta X_{i}}{\overset{\wedge }{P}(X^{*})}\left({\frac{\partial \overset{\wedge }{P}}{\partial X_{i}}|}_{X^{*}}\right)\times 100\%,$$

where $X^{*}=(X_{1}^{*},X_{2}^{*},...)$ are the values of the parameters given in Table 1 and $\Delta X_{i}=0.1X_{1}^{*}$. The obtained values represent the percentage-wise increase in $\overset{\wedge }{P}$ if a parameter increases by 10%, assuming the relationship can be approximated as linear for this small change. The results are given in Table 2. In the second approach, we assign all, or all but one of the parameters the values from Table 1:

(57) **X **= X*,

or

(58) $$X=X_{i}^{*}\equiv (X_{1}^{*},...,X_{i},X_{i+1}^{*},...),$$

respectively, and then numerically solve the equations

(59) $$q\overset{\wedge }{P}(X^{*})=\overset{\wedge }{P}(X_{i}^{*})$$

for each *X*_*i*_, where *q *= 1.25 or *q *= 1.5. The results are given in Table 3.
